# “From Surface to Substrate: Advancing VT Ablation Strategies for Deep Arrhythmic Substrates”—A Review of Emerging Techniques in Intramural Lesion Formation

**DOI:** 10.31083/RCM47993

**Published:** 2026-05-26

**Authors:** Krittapoom Akrawinthawong, Roopesh Sai Jakulla, Takumi Yamada

**Affiliations:** ^1^Section of Cardiac Electrophysiology, University of Oklahoma Health Science Center, Oklahoma City, OK 73104, USA; ^2^Cardiovascular Division, University of Minnesota, Minneapolis, MN 55455, USA

**Keywords:** ventricular tachyarrhythmias, ventricular tachycardia, radiofrequency ablation, catheters

## Abstract

Intramural ventricular tachycardia represents a formidable frontier in catheter ablation, where the arrhythmogenic substrate resides deep within the myocardial wall, often beyond the reach of conventional energy delivery. Achieving durable lesion formation in this setting requires a strategic balance between transmural efficacy and procedural safety. This review synthesizes the evolving landscape of intramural ventricular tachycardia ablation, detailing contemporary techniques, including bipolar ablation, needle-based approaches, simultaneous endocardial–epicardial delivery, and emerging energy modalities, each tailored to overcome the limitations of lesion depth and tissue heterogeneity. We examine diagnostic criteria, imaging adjuncts, and electroanatomic mapping strategies that guide procedural planning and highlight innovations aimed at enhancing lesion depth without compromising adjacent structures. By integrating current evidence and expert practice, this article offers a comprehensive framework for navigating the complexities of intramural ventricular tachycardia ablation and advancing outcomes in this challenging domain.

## 1. Introduction

Since the landmark report of successful percutaneous catheter ablation for arrhythmia [1], this technique has become a cornerstone in the management of a wide array of cardiac rhythm disorders. The efficacy of catheter ablation is fundamentally tied to the durability of lesion formation, which must be carefully balanced against the risk of collateral injury from energy delivery. Over the past four decades, relentless innovation in ablation technologies and catheter design has propelled the field forward at an extraordinary pace [2]. Among the most challenging ablation target is ventricular tachycardia (VT), where the arrhythmic substrates often span complex three-dimensional pathways and variable myocardial depths [3, 4].

The diagnostic criteria for intramural ventricular arrhythmias (VAs) have been delineated in the previous reports [5, 6, 7]. These criteria typically integrate electroanatomic mapping findings, imaging modalities, and ablation response characteristics to identify arrhythmogenic foci located within the mid-myocardial layers (Table 1). These include the absence of early activation signals on both endocardial and epicardial surfaces, poorly matched pace map correlation from either side, and successful ablation only with deep or bipolar energy delivery—suggesting a mid-myocardial origin. Imaging modalities such as cardiac magnetic resonance imaging (MRI) and computed tomography (CT) further support the diagnosis by revealing scar confined to the intramural layers. Collectively, these criteria help guide targeted ablation strategies for intramural VAs.

**Table 1.** **Criteria of intramural ventricular arrhythmia**.

Criterion	Electrophysiologic interpretation
Electrophysiological and 3D mapping criteria	
1. Local ventricular activation <–10 ms pre-QRS on both 2 opposing cardiac chambers	Suggest activation occurs deeper in the myocardial wall
2. The earliest local ventricular activation <–20 ms pre-QRS on either 2 opposing cardiac chambers	Earliest signal should localize to one surface; insufficient earliness suggests an origin distant from 2 opposing sites.
3. Far-field ventricular pre-potential recorded at both endocardial and epicardial sides or opposing cardiac chambers during ventricular arrhythmia	Electrograms suggest that the true origin is deeper than the recording electrodes, as in cases of LVOT or intraseptal VA.
4. Relatively wide areas of early activation on myocardial surfaces or absence of QS unipolar electrogram at the earliest activation site	Activation propagates outward from mid-myocardium
5. Suboptimal pace map from both endocardium and epicardium and intramural pace maps show better correlation than unipolar pace map from myocardial surfaces	Pacing fails to reproduce VA morphology despite adequate output, indicating the site is not arrhythmia origin
6. Recorded local ventricular activation from intramural cardiac venous branches is earlier than any endocardial or epicardial sites	Confirm arrhythmia origin within intramural myocardial tissue
Ablation criteria	
1. Unsuccessful RF ablation at the sites of the earliest local ventricular activation/best pace map, with either successful sequential, simultaneous or bipolar RF ablation at the sites between 2 chambers	Standard ablation’s lesion fails to reach the intramural substrate
2. Transient suppression of VAs during RF ablation from either endocardial or epicardial side or from 2 opposing cardiac chambers	Partial effect indicates energy delivery affects but does not fully eliminate the intramural arrhythmia focus

RF, radiofrequency; VA, ventricular arrhythmia; LVOT, left ventricular outflow tract.

For intramural VAs, conventional thermal energy may fail to reach the critical tissue layers necessary for effective ablation. The concept of transmurality—achieving full-thickness myocardial injury—remains central to VT ablation and is a key determinant of procedural success. This review explores current strategies, energy modalities, and emerging technologies designed to enhance lesion formation during cardiac ablation, with an emphasis on optimizing both procedural efficacy and patient safety.

## 2. Optimizing Lesion Formation Through Biophysical Control

Effective catheter ablation for VT necessitates precise modulation of lesion depth to eliminate deep-seated arrhythmogenic substrates while minimizing injury to adjacent structures. Achieving this balance requires a comprehensive understanding of the biophysical principles governing energy delivery. Radiofrequency (RF) energy remains the most widely utilized modality [8], producing myocardial necrosis through thermal injury generated by alternating current (~500 kHz) between the catheter tip (anode) and a dispersive ground patch (cathode). Heat is primarily generated via resistive heating at the electrode–tissue interface, followed by conductive thermal spread into deeper myocardial layers. Irreversible tissue injury typically occurs at temperatures exceeding 50 °C [9, 10]. Optimizing lesion formation involves several factors such as fine-tuning several procedural parameters, including contact force (CF), duration of energy delivery, catheter stability, power output, and both tissue and surrounding impedance.

Among those factors, CF—measured at the catheter tip—has emerged as a critical determinant of lesion size and depth [11]. Elevated CF enhances energy transfer efficiency but also increases the risk of steam pops and thrombus formation [12]. Adequate CF ensures stable catheter–tissue coupling, which is essential for consistent lesion formation [13]. A CF range of 10 to 40 grams, varying by anatomical location, is recommended to balance efficacy and safety [14, 15, 16]. The introduction of real-time CF monitoring has significantly improved procedural reproducibility and reduced complication rates in both animal and human studies [17, 18]. Furthermore, positive CF during a diastole has been associated with a higher yield of late potential detection within scarred myocardium, underscoring its importance in substrate mapping. Optimal CF thresholds for mapping have been reported as 9 grams for the right ventricle, 8 grams for the left ventricle, and 8 grams for epicardial surfaces [19, 20].

Expanding on CF, the force-time integral (FTI) combines the magnitude of contact force with the duration of energy application, providing a cumulative measure of mechanical engagement. Expressed in gram-seconds (gs), FTI has been correlated with lesion durability, with values exceeding 400 gs associated with effective lesion formation [21, 22]. However, its inability to incorporate power delivery limits its predictive accuracy, particularly in heterogeneous or fibrotic substrates of VT circuit. Despite this limitation, FTI remains a valuable intra-procedural guide and has been validated in both preclinical and clinical settings, primarily in atrial fibrillation ablation [23, 24].

To address the shortcomings of FTI, the ablation index (AI) has been developed as a composite metric integrating contact force, application duration, and RF power into a weighted formula [25, 26]. AI offers a more robust prediction of lesion quality and has been widely adopted in workflows for pulmonary vein isolation [27]. Its use in VT ablation is expanding [28, 29], particularly in anatomically complex regions where lesion reproducibility is paramount. *In vitro* studies have demonstrated AI’s superior correlation with lesion depth compared to FTI, and clinical trials have validated its utility in guiding ablation strategy and improving outcomes [30, 31].

Additional CF-related metrics, such as force vector analysis, may further refine lesion targeting while minimizing collateral damage to adjacent structures like the lungs, coronary arteries, and phrenic nerve during epicardial VT ablation [32]. Notably, much of the clinical data guiding CF and its derivatives in VT ablation has been extrapolated from atrial fibrillation studies. This underscores the need for dedicated investigations tailored specifically to VT ablation [33, 34], to optimize safety and efficacy in this distinct arrhythmogenic context. Other biophysical parameters, such as impedance, should also be considered, particularly in light of emerging technologies that focus on dedicated impedance monitoring. Unlike global impedance, Boston Scientific’s DirectSense™ measures tissue resistivity directly at the catheter tip, providing real-time feedback on subsurface heating and lesion formation. A local impedance drop correlates with effective lesion creation, which is especially important in thick ventricular myocardium. This technology enables operators to optimize energy delivery, avoid ineffective ablation, and minimize collateral damage, but additional evidence is needed to fully establish its clinical value [35, 36].

## 3. Energy Delivery Strategies

### 3.1 Simultaneous and Sequential Unipolar Ablation

Simultaneous unipolar ablation is designed to counteract the effects of convective cooling caused by high blood flow at endocardial or epicardial sites, thereby facilitating thermal elevation within intramural myocardial regions. This technique enhances the efficacy of ablating deep arrhythmogenic substrates by minimizing the heat-sink effect of the surrounding myocardium and expanding the zone of conductive heating around each lesion created by the ablation catheter [5, 37]. The experimental study demonstrated that a simultaneous unipolar RF lesion was larger than twice the size of a single electrode RF lesion while the tissue temperature between the two electrodes was higher than that at the same distance from the single electrode [37]. Operationally, this method employs two independent RF generators, allowing individualized power modulation and impedance monitoring for each catheter. This configuration enables optimal lesion formation even in the presence of impedance mismatch between opposing sites such as the sites within the great cardiac vein and endocardial left ventricular outflow tract. Importantly, RF energy delivery must be initiated at 0 Watts to prevent generator error messages during simultaneous activation [38].

**Fig. 1.**
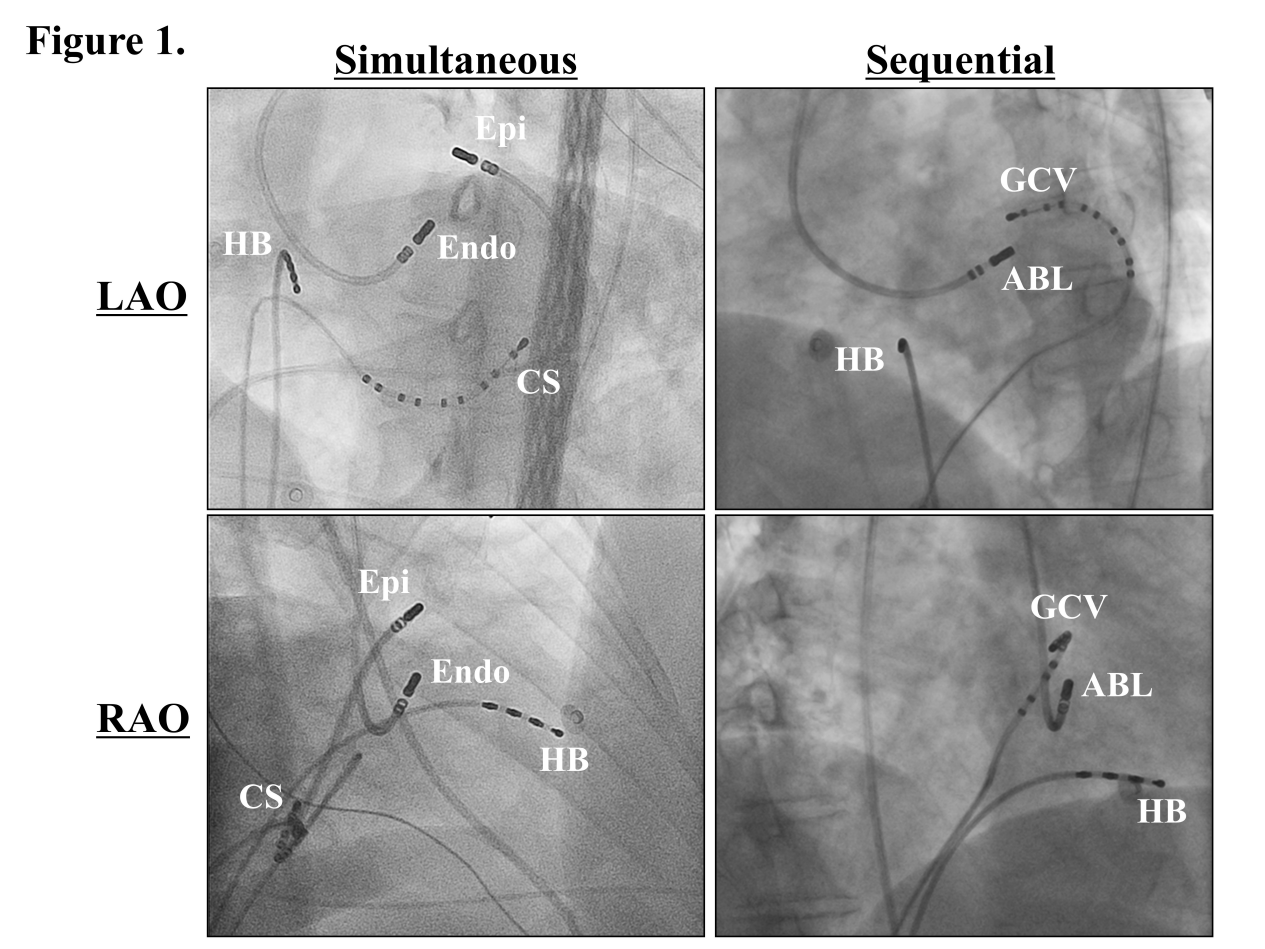
**Fluoroscopic images exhibiting the ablation sites**. ABL, the ablation catheter; CS, coronary sinus; Endo, the endocardial ablation catheter; Epi, the epicardial ablation catheter positioned within the great cardiac vein (GCV); HB, His bundle; LAO, left anterior oblique view; RAO, right anterior oblique view. This figure was cited from reference [7] with permission.

**Fig. 2.**
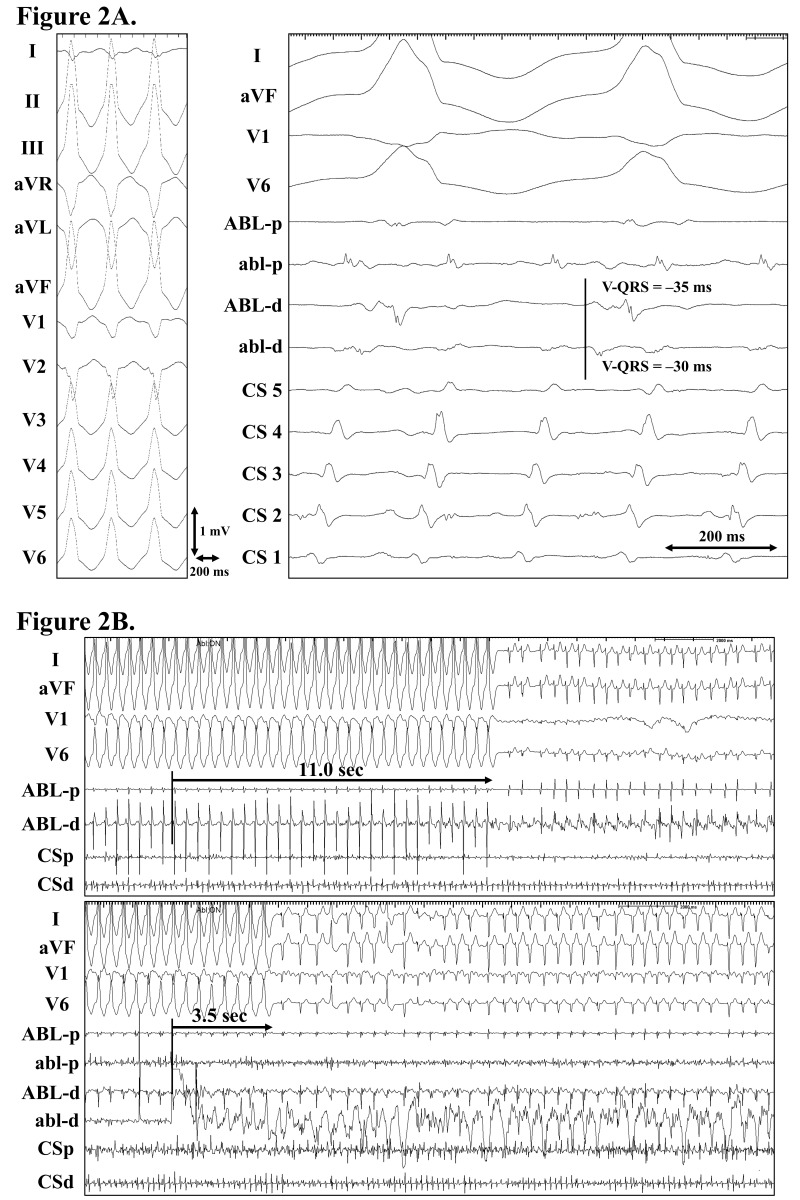
**Idiopathic intramural left ventricular outflow tachycardia successfully eliminated by a simultaneous unipolar radiofrequency ablation from the endocardial and epicardial sites**. (A) Electrocardiograms exhibiting the ventricular tachycardia (left panel) and cardiac tracings exhibiting the successful ablation sites (right panel). Note that the patient was also in atrial flutter. A far-field ventricular pre-potential was recorded from the ablation catheter positioned within the GCV (ABL-d). (B) Cardiac tracings exhibiting the effective (upper panel) and successful ablations (lower panel). An effective ablation was achieved by a radiofrequency ablation within the GCV, and the successful ablation was achieved by a simultaneous unipolar radiofrequency ablation from the GCV and aorto-mitral continuity (AMC). Note that the ventricular tachycardia terminated more quickly during the successful ablation than the effective ablation. ABL (abl), the ablation catheter positioned within the GCV (AMC); CS 1 to 5, the first (most distal) to fifth (most proximal) electrode pairs of the CS catheter; V-QRS, the local ventricular activation time relative to the QRS onset. The other abbreviations are as in Fig. 1. These figures were cited from reference [7] with permission.

Clinical applications have demonstrated promising outcomes. Yamada *et al*. [6, 7] reported the successful use of simultaneous unipolar ablation in patients with intramural left ventricular outflow tract VAs, particularly after failed sequential unipolar attempts in 5 of 14 cases (Figs. 1,2, Ref. [7]). Their analysis indicated that simultaneous ablation was most effective when the anatomical distance between endocardial and epicardial targets exceeded 8 mm and when the earliest local ventricular activation preceded QRS onset by less than 30 milliseconds. In a separate case series involving six patients with non-ischemic cardiomyopathy and mid-myocardial substrate VT, prolonged sequential unipolar ablation terminated VT in 3 of 4 patients whose VT was ongoing, suggesting transient efficacy due to insufficient lesion depth. However, subsequent simultaneous unipolar ablation achieved complete VT elimination and non-inducibility in all cases, without steam pops—likely reflecting deeper lesion formation within the mid-myocardial substrate [38].

Overall, this technique is readily implementable in standard electrophysiology laboratories, requiring only the availability of two RF generators. The use of dual irrigated catheters with independent power and impedance control underscores both the practicality and safety of this approach.

### 3.2 Bipolar Ablation

Optimizing lesion formation during ablation requires minimizing energy dissipation through low-resistance pathways, thereby enhancing current delivery to the target tissue. A dual-electrode configuration—comprising one active and one return electrode—was first investigated over three decades ago as a means to improve lesion depth and volume [37]. By excluding low-resistance extraneous elements such as grounding pads, blood, and surrounding tissue from the ablation circuit, and instead concentrating current flow between two closely positioned electrodes, the efficiency of energy transfer can be significantly increased. Foundational *in vitro* studies [37, 39] validated this principle, demonstrating that bipolar electrode configurations produced lesions nearly twice the size of those generated by sequential unipolar setups. Subsequent experimental investigations [40, 41] have consistently corroborated these findings, and the technique has since been translated into clinical practice with promising outcomes.

Notably, the use of 0.45% saline irrigation did not significantly influence lesion characteristics in well-optimized bipolar ablation configurations [42]. Prolonged energy delivery—specifically, 120 seconds at 30 Watts—has been shown to achieve transmural lesions with enhanced safety, reducing the incidence of steam pops without compromising lesion depth compared to higher power settings [42]. To date, the influence of contact force using commercially available catheters remains unexplored, as most studies have employed a perpendicular electrode orientation. This configuration may not be feasible in anatomically constrained regions such as the pericardium. Recent *ex vivo* investigations have highlighted the advantages of a “true parallel configuration”, wherein both active and return electrodes are aligned parallel to the myocardial surface. This setup demonstrated the most favorable safety profile, with the lowest incidence of steam pops during bipolar ablation [43]. The active electrode consistently exhibits greater temperature elevation and contributes more substantially to lesion formation [44]; thus, it should be executed with an irrigated ablation catheter and positioned on the side with lower thromboembolic risk—typically the right heart or epicardial surface. Lesion transmurality appears to be primarily governed by ablation duration, whereas steam pop risk is more closely associated with tissue thickness and power selection [40, 41, 43]. Furthermore, increased power correlates with greater lesion volume irrespective of electrode orientation [43]. The efficacy of bipolar ablation is theoretically diminished when the interelectrode distance increases, underscoring the importance of spatial proximity for optimal energy delivery [43].

**Fig. 3.**
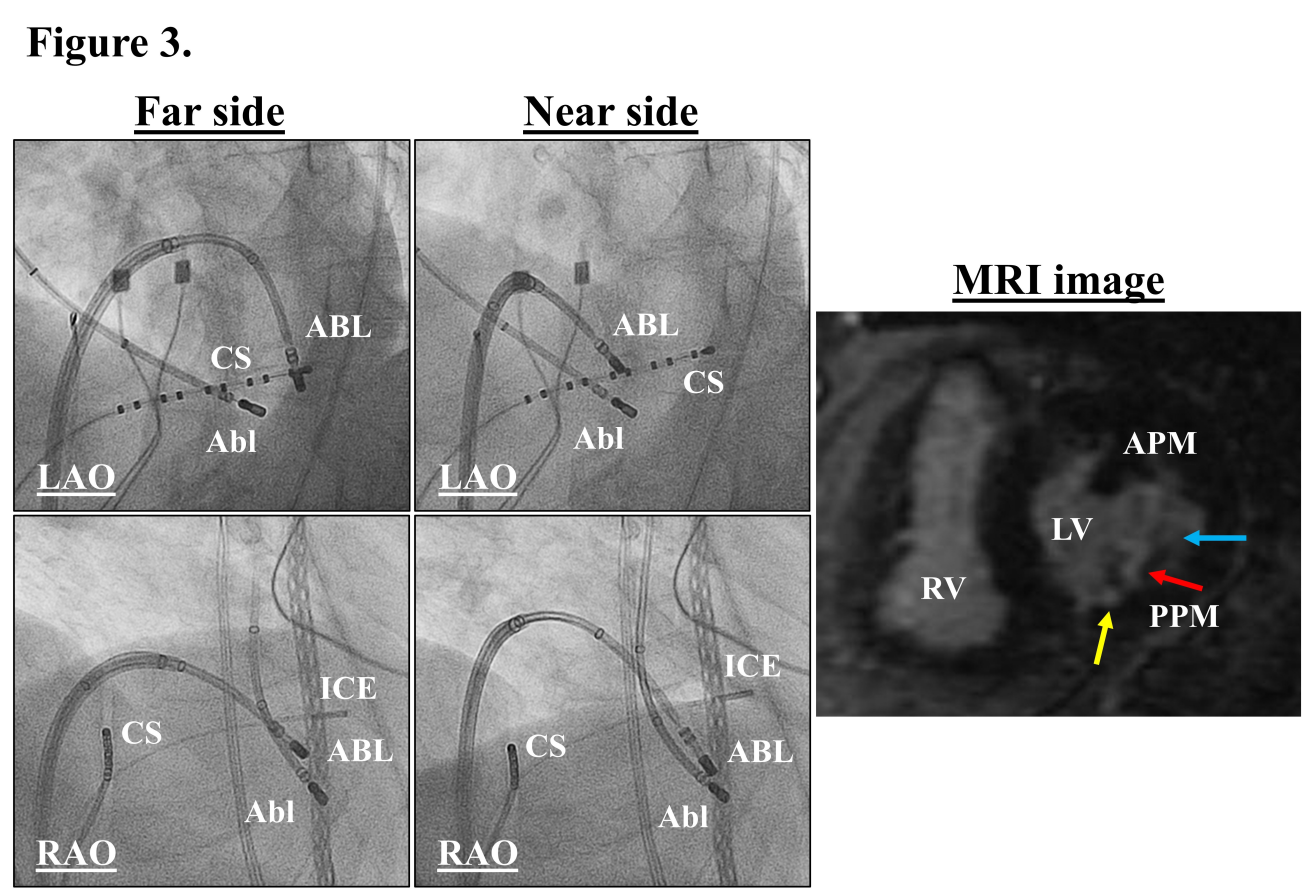
**Fluoroscopic images exhibiting the unsuccessful (left panels) and successful bipolar ablation sites (middle panels) and late Gadolinium-enhanced cardiac magnetic resonance image exhibiting the radiofrequency lesions on the posterior papillary muscle (PPM) in the left ventricle (right panel)**. The yellow, blue, and red arrows indicate deeper scar on the septal and lateral sides, and the deepest scar in the middle of the PPM. Abl, transaortic ablation catheter; APM, anterior papillary muscle; ICE, intracardiac echocardiographic catheter; LV, left ventricle; RV, right ventricle. The other abbreviations are as in Fig. 1. This figure was cited from reference [43] with permission.

**Fig. 4.**
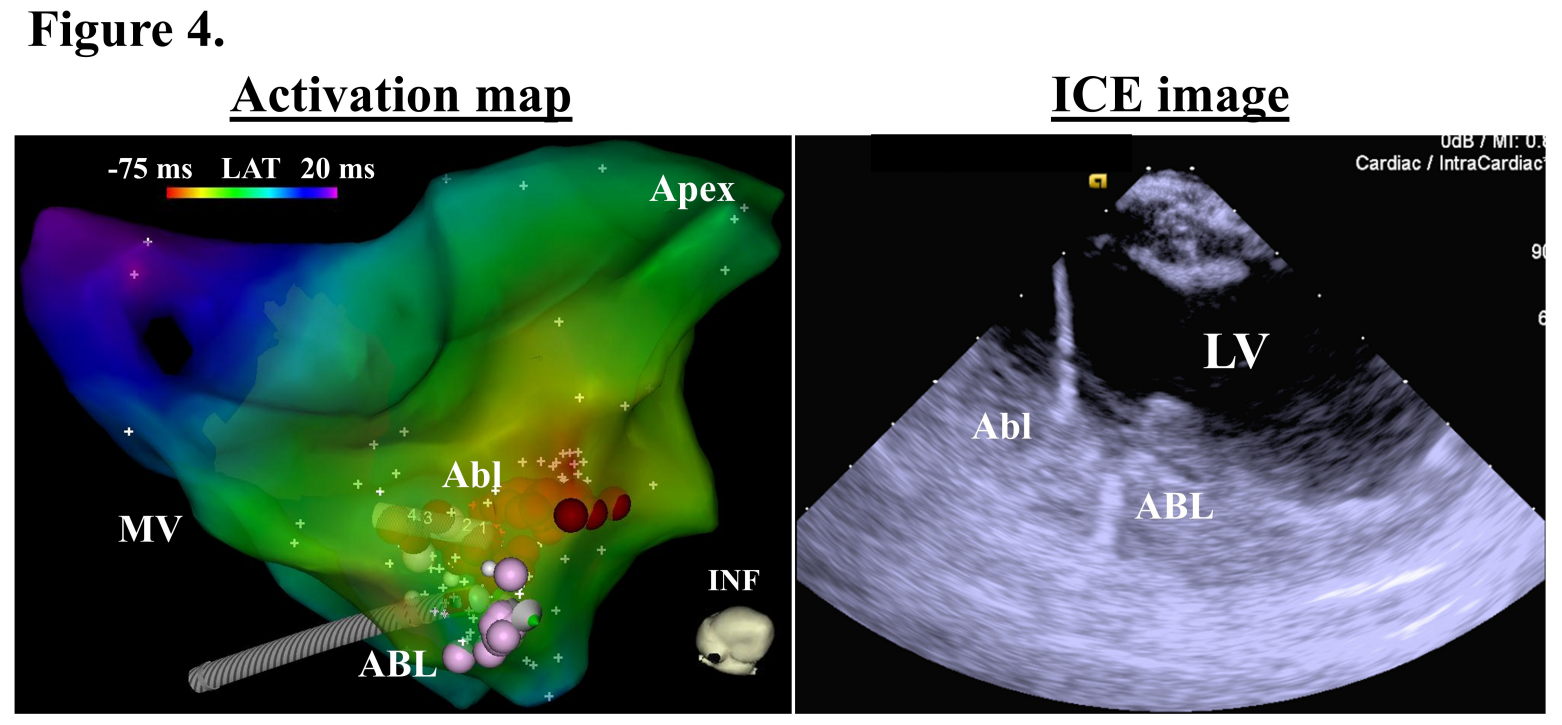
**An activation map (left panel) and intracardiac echocardiographic (ICE) image (right panel) exhibiting the successful ablation site**. The red and pink tags in the activation map indicate the ablation sites. INF, inferior; LAT, local activation time; MV, mitral valve. The other abbreviations are as in the previous figures. This figure was cited from reference [43] with permission.

Clinical application of bipolar ablation has predominantly targeted anatomical regions where VA’s substrates are suspected to reside intramurally. These include the intramural ventricular outflow tract—utilizing an active electrode positioned at the left pulmonic cusp via a reverse-U maneuver and a return electrode in the left ventricular outflow tract—and the interventricular septum, with the active electrode placed in the right ventricle and the return electrode in the left ventricle [44] and the papillary muscle with two electrodes placed on both sides [45] (Figs. 3,4, Ref. [43]).

It is imperative to consider impedance mismatches between electrode sites and to appropriately balance electrode sizes, as lesions tend to form preferentially around smaller electrodes due to elevated current density. Traditional electrogram-based targeting strategies—such as unipolar and bipolar electrogram’s characters—may not reliably identify optimal sites for bipolar ablation. Instead, transient suppression observed during prior unipolar ablation may serve as a valuable indicator for selecting this alternative approach following failure of unipolar ablation [44].

Pre-procedural assessment of adjacent anatomical structures remains critical to mitigate the risk of collateral injury. This includes coronary angiography and precise localization of the His bundle signal prior to energy delivery [46, 47, 48]. A comprehensive understanding of the variables influencing lesion formation—such as power settings, duration of energy application, catheter tip size and type, electrode orientation, and interelectrode spacing—is essential for maximizing procedural efficacy while maintaining safety. When appropriately selected and executed, bipolar ablation offers a promising strategy for the effective elimination of intramural VAs without compromising procedural safety.

### 3.3 Multipolar Radiofrequency Ablation

Multipolar radiofrequency ablation (MPA) emerged as a solution to the limitations inherent in bipolar ablation, particularly in cases where intramural VT circuits reside in regions inaccessible to precise positioning of the return electrode. By integrating electrodes from a multipolar mapping catheter into the ablation circuit as return electrodes, operators gain the ability to fine-tune lesion delivery with enhanced spatial control. Fernandes *et al*. [49] first demonstrated the feasibility of this approach, showing *ex vivo* lesion formation and successful application in a clinical setting. Deployment of a 2F multipolar catheter within the coronary venous system enables targeted ablation in anatomically challenging or otherwise unreachable regions. The flexibility in selecting return electrodes on the mapping catheter allows for precise lesion placement while minimizing collateral damage to adjacent coronary arteries. Notably, the smaller surface area of these electrodes increases local current density, facilitating the creation of larger lesions with reduced power requirements.

In a pioneering clinical series involving 31 patients undergoing ablation for ventricular arrhythmias, MPA demonstrated efficacy across complex anatomical substrates such as the left ventricular summit, cardiac crux, and interventricular septum [50]. Remarkably, when MPA was employed without prior RF energy delivery during the same index procedure, an acute success rate of 100% was observed—likely attributable to the absence of tissue edema that can hinder lesion formation. However, the occurrence of char formation on return electrodes underscores the importance of positioning the multipolar catheter within epicardial spaces or right-sided cardiac chambers to mitigate the risk of systemic thromboembolism. Adjunctive strategies, such as saline infusion or incorporation of a dispersive patch to reduce circuit impedance, may further optimize procedural safety and efficacy.

Despite its promise, further investigation is warranted to elucidate the influence of contact force and local impedance at the microelectrode-tissue interface on lesion characteristics. Continued advancements in mapping catheter technology are poised to enhance the utility and adoption of MPA, offering a streamlined setup and obviating the need for high-risk maneuvers such as epicardial access.

### 3.4 Cryoenergy for VT Ablation

Cryoablation induces cellular injury through rapid freezing, resulting in intracellular ice crystal formation and disruption of microvascular integrity. This process leads to irreversible cell death, with subsequent replacement by fibrotic tissue over time [51]. Owing to its relatively controlled and localized thermal effects, cryoenergy has been considered a more tissue-sparing modality compared to RF ablation in general. Consequently, it is often employed in regions adjacent to critical anatomical structures such as the His bundle or coronary vasculature, where precision and safety are paramount.

In preclinical canine models, cryoablation at –75 °C within the coronary sinus has demonstrated the ability to produce transmural lesions comparable to those achieved with RF energy, but with a significantly reduced risk of coronary artery stenosis when the vessel lies within 2 mm of the ablation site [52]. This safety profile has been corroborated in clinical settings, including successful application of cryoablation in the right ventricular outflow tract and left ventricular summit near coronary arteries [53].

Lesion formation during cryoablation is influenced by conventional parameters similar to those governing RF ablation, including energy delivery duration, temperature, and electrode size but is not by a local impedance. For example, RF energy delivery may be limited within the cardiac venous system owing to a high impedance whereas cryoenergy can be delivered irrespective of the local impedance. A distinctive feature of cryoablation is its capacity to enhance tissue thermal conductivity through repetitive freeze–thaw cycles, although the underlying mechanisms remain incompletely understood. This property has led to the hypothesis that sequential application of cryoablation followed by RF energy may augment lesion depth and transmurality. Cryoablation is thought to modify tissue architecture by reducing impedance and altering cellular integrity, thereby facilitating more uniform and deeper RF energy propagation—particularly in thick or fibrotic myocardial substrates. Despite promising preclinical observations, this synergistic approach has not yet been substantiated by robust clinical evidence and remains investigational in human subjects. Additionally, cryoablation exhibits a phenomenon known as cryotermination or cryomapping, characterized by progressive slowing of electrical conduction culminating in reversible block at sub-lethal temperatures. This effect offers a strategic advantage for mapping focal VAs with minimal tissue injury [51, 54].

Recent advancements in catheter technology have introduced platforms capable of delivering cryothermal energy specifically for VA ablation. Among these, ultralow-temperature cryoablation (ULTC) utilizing near-critical nitrogen at –196 °C has gained traction due to its ability to produce deep lesions ranging from 4 to 10 mm [55]. This modality was evaluated in a single-arm, first-in-human study—Cryocure-VT—which demonstrated an acute procedural success rate of 94% in this study enrolling patients with either ischemic or non-ischemic cardiomyopathies, defined by non-inducibility of clinical VT. At six months post-ablation, 60.3% of patients remained free from VT recurrence [55]. Building on these promising results, a larger prospective, multi-center pivotal trial—FULCRUM-VT (NCT05675865)—is currently underway. This study includes 206 patients with ischemic or non-ischemic monomorphic VT and spans 19 clinical sites. The outcomes of this trial are anticipated to further elucidate the safety and efficacy of ULTC in the treatment of VAs with complex substrates.

## 4. Irrigation Medium and Conductivity Modulation

### 4.1 Half-Normal Saline Irrigation

Half-normal saline (HNS) irrigation has been proposed to enhance RF current delivery into targeted myocardial tissue by minimizing energy dissipation into the surrounding lower-impedance environment [56]. Due to its reduced ionic concentration, HNS creates a higher impedance milieu at the catheter–tissue interface, thereby promoting deeper and more focused lesion formation. Building on this principle, dextrose water has demonstrated the potential to generate even larger lesions compared to HNS and normal saline when used as an irrigant. However, its application is associated with increased risk of steam pops, attributed to unstable impedance dynamics and challenges in real-time monitoring [57].

It is important to note that the efficacy of HNS irrigation may be context dependent. Its benefits are most pronounced in anatomical settings devoid of competing low-impedance flows—such as the epicardial space or small vascular structures—where catheter orientation and contact are critical. High contact force may further augment the tissue–electrode interface, potentiating lesion depth and consistency.

This irrigation strategy can be integrated as an adjunctive or bail-out technique alongside other advanced ablation modalities, including simultaneous unipolar or bipolar ablation, to optimize lesion expansion. Nonetheless, its clinical utility remains uncertain, as existing data are limited, variably reproducible, and largely derived from preclinical models [56, 57]. Further *in vivo* and human studies are warranted to validate its safety and efficacy.

### 4.2 Ablation Ground Patch Modification

Optimizing the location of the dispersive ground patch is a logical strategy to minimize RF energy loss and enhance current delivery through the targeted myocardial tissue. Traditionally, the ground patch is affixed to the patient’s thigh or back in cardiac electrophysiology procedures. However, repositioning can be performed with minimal difficulty and may significantly increase lesion size at the ablation site [58]. This adjustment may also contribute to a lower baseline impedance, which has been associated with increased current output and larger lesion dimensions during power-controlled ablation [59]. Since RF current output is influenced by impedance load, strategic patch placement becomes a critical biophysical consideration.

A recent *in vivo* study demonstrated that orienting the ground patch in a “concordant” direction relative to the ablation vector can increase lesion depth by approximately 20% [60]. For instance, when ablating the anterior right ventricular outflow tract, positioning the ground patch on the anterior chest wall is both practical and effective. A pragmatic approach involves placing patches on both anterior and posterior aspects of the torso at the outset of the procedure, allowing for selection based on the identified ablation target. This method is cost-effective and easily implementable.

Nevertheless, anatomical variability among patients necessitates individualized assessment. There is no universally optimal ground patch location, and successful implementation of this strategy requires careful consideration of biophysical parameters and patient-specific anatomy.

## 5. Novel Approaches in VT Ablation

Given the inherent limitations of RF energy—particularly regarding lesion depth and safety—alternative energy modalities and ablation techniques have been concurrently developed to enhance efficacy in VT ablation. Each approach offers distinct advantages and drawbacks, allowing operators to tailor their strategy based on the specific anatomical and electrophysiological characteristics of the arrhythmogenic substrate. This individualized selection aims to optimize procedural success while minimizing collateral injury.

### 5.1 Venous and Arterial Ethanol Ablation 

Ethanol, a potent cytotoxic agent, is administered via venous or arterial infusion to induce targeted myocardial injury. The pathophysiological mechanisms underlying ethanol-induced cellular damage include endothelial disruption, protein denaturation, inflammatory response, and direct endothelial injury [61]. The earliest documented use of this technique dates back to 1988, when cellular necrosis was achieved in two patients with ischemic VT through ethanol infusion [62]. This pioneering report also introduced the concept of mapping the ablation site by temporarily disrupting local perfusion using cold normal saline prior to ethanol delivery, thereby predicting the area of permanent injury.

**Fig. 5.**
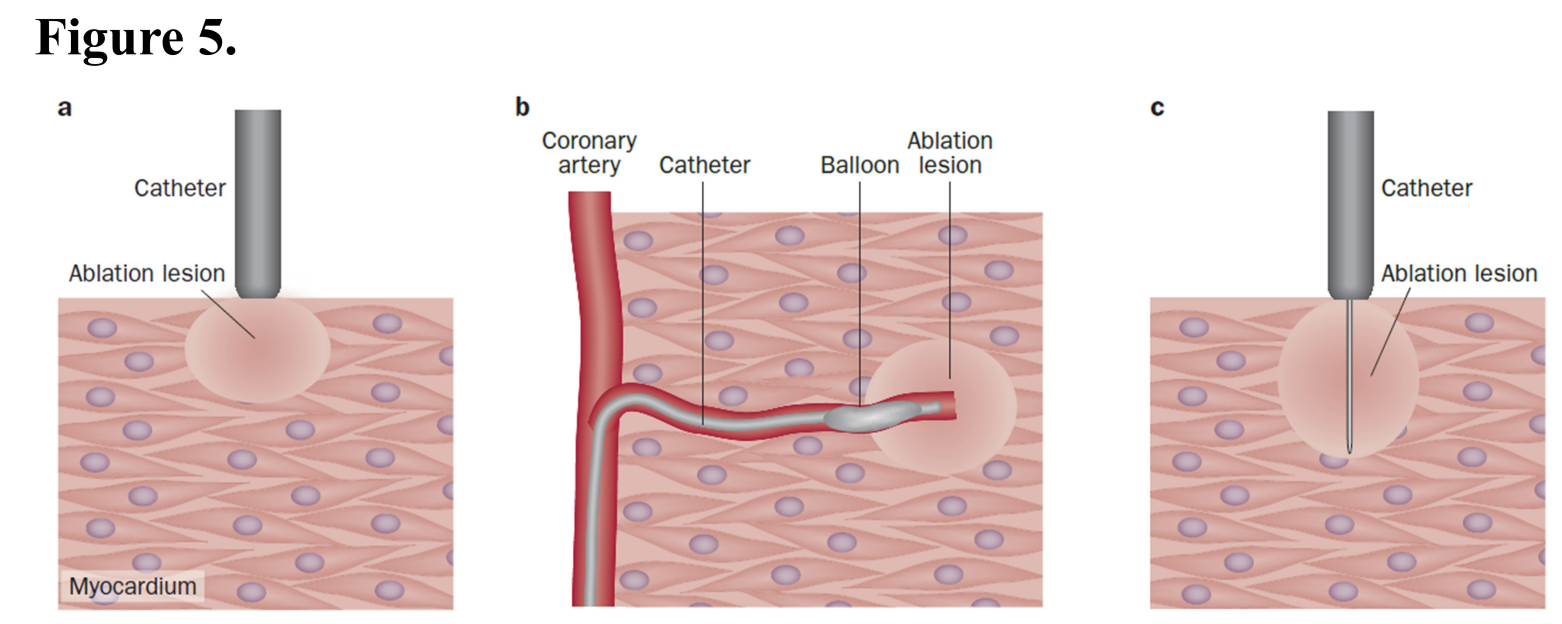
**Ablation strategies for ventricular tachycardias**. (a) In radiofrequency ablation, the lesion is created underneath the tip of the catheter and does not reach the deep myocardium below the surface. (b) Transcoronary ethanol ablation can be used to create a lesion deep inside the myocardium, although the location of the lesion is limited by the distribution of the coronary artery branches. (c) In needle ablation, the lesion created underneath the tip of the catheter expands to the tip of the needle, which can be located deep inside the myocardium. This figure was cited from reference [65] with permission.

With advancements in catheter design and coronary angiography techniques, ethanol ablation has gained traction among cardiac electrophysiologists. However, the non-selective cytotoxicity of ethanol necessitates meticulous delivery and containment. Inadvertent ethanol dispersion or imprecise catheter placement may result in off-target myocardial damage, pericardial effusion, cardiac tamponade, or systemic embolization [63, 64]. Therefore, procedural refinement should prioritize precision targeting and containment—often achieved through balloon occlusion—to maximize efficacy and minimize collateral injury (Fig. 5, Ref. [65]) [65, 66, 67]. Ethanol leakage into adjacent healthy myocardium may provoke novel arrhythmogenic substrates or compromise critical structures such as the cardiac conduction system.

Although arterial ethanol infusion was the initially developed approach, the retrograde venous technique has emerged as a preferred alternative due to its reduced risk of coronary injury and decreased reliance on interventional cardiology support. This method has been extensively described in the literature [63, 68, 69, 70, 71]. Meta-analytic data suggest that the anterograde transarterial route is associated with higher rates of ventricular arrhythmia recurrence and procedural complications compared to the retrograde transvenous approach, with anatomical or technical limitations impeding acute success in approximately 30% of cases [72].

Ethanol ablation is applicable to both coronary venous and arterial structures, including the septal perforator and posterior descending artery [73]. Mechanistically, retrograde venous injections induce direct myocardial and capillary injury, whereas arterial ethanol infusion primarily achieves ablation through coronary occlusion [66, 74, 75]. Given the procedural complexity, coronary artery cannulation should be reserved for experienced operators. Vessel caliber must be assessed intra-procedurally to determine the appropriate ethanol volume required for effective lesion formation. Targeting smaller-caliber vessels and employing balloon occlusion can help confine ethanol distribution, thereby reducing unintended tissue damage. Pre-injection visualization of the vascular territory is essential to anticipate potential injury to adjacent critical structures.

Integration of ethanol ablation with electrophysiological mapping—using an angioplasty wire connected via alligator clips—has proven to be a pragmatic strategy [76]. This approach enables precise localization of arrhythmogenic myocardium through pacing or activation mapping prior to ablation. Kreidieh *et al*. [77] reported a case series involving seven patients with refractory VAs originating from the left ventricular summit. Ablation via the septal and lateral left ventricular veins yielded a 100% acute success rate without complications; however, VT recurred in four patients within 1–2 years [77]. The authors proposed this method as a salvage strategy for anatomically challenging arrhythmias where venous anatomy permits ethanol infusion. Their findings demonstrated obliteration of postcapillary perfusion alongside augmentation of precapillary collateral flow, a phenomenon that may underlie the myocardial injury responsible for arrhythmia suppression in this ablation technique. A comprehensive understanding of coronary venous anatomy is thus imperative for procedural success [65, 78].

Despite its promise, several unresolved questions remain. It may be challenging to control a lesion size created by ethanol infusion. The double-balloon technique enables precise lesion targeting by isolating a coronary vein segment overlying the VT substrate. Two occlusion balloons are deployed—one proximally to obstruct retrograde flow and one distally to prevent antegrade perfusion into collateral branches. Ethanol is infused into the sealed segment, allowing transmural diffusion through the vein wall to ablate the underlying myocardium [71]. Optimal ethanol dosing—both in terms of volume and number of injections—requires further investigation to minimize long-term recurrence and collateral injury. Additionally, strategies to prevent revascularization of the ablated substrate are needed. Incorporating ethanol ablation as a bailout technique [77] or as a sole modality [79] for VAs in anatomically inaccessible regions offers a valuable adjunct to conventional energy-based ablation methods such as RF energy or cryotherapy.

### 5.2 Pulsed-Field Ablation (PFA)

PFA is an emerging nonthermal energy modality that employs high-voltage, ultra-short electrical pulses—typically in the nanosecond range—to induce irreversible electroporation of cardiac tissue [80, 81]. Unlike traditional thermal ablation, PFA preferentially disrupts cellular membranes through electric field-mediated effects targeting intracellular organelles, resulting in rapid lesion formation with minimal collateral damage.

The biophysical determinants of lesion formation and durability include electric field strength, pulse number and repetition rate, pulse shape and duration, as well as electrode size, spacing, contact force, and orientation [82, 83, 84]. Bipolar energy delivery is commonly utilized due to its localized field distribution, which reduces musculoskeletal stimulation and procedural discomfort. The myocardial selectivity of PFA may be attributed to the relatively larger diameter of cardiomyocytes compared to smooth muscle and neural cells, enhancing safety and precision in targeting cardiac tissue [85].

In the context of VT ablation, several issues remain under consideration [85, 86, 87]. First, the structural substrate of VT is inherently complex, often comprising heterogeneous tissues such as adipose and fibrotic scars. Recent studies have shown that PFA can effectively produce deep lesions in fibrotic substrates, including infarcted and RF-induced scar tissue, with lesion characteristics comparable to those in healthy myocardium. In animal models, lesion depths reached up to 8.6 mm, with potential for epicardial extension and transmurality. Nonetheless, further clinical research is required to elucidate the effects of PFA energy on well-matured scar tissue under variable contact forces, aiming to balance efficacy and safety in human applications [88]. Second, myocardial fiber orientation relative to the electric field is critical; the layered and multidirectional architecture of ventricular myocardium may influence lesion transmurality. Third, while Joule heating is generally negligible, higher field strengths necessary for VT ablation —aimed at achieving deeper, transmural lesions—can enhance tissue conductivity as temperature rises, potentially amplifying thermal effects and increasing the risk of collateral injury. Additionally, although apoptosis is the predominant mechanism of cell death in PFA, necrosis may also contribute under certain conditions. PFA has been linked to coronary artery spasm, particularly when delivered near coronary vessels. Proposed mechanisms include autonomic activation, vascular smooth muscle stimulation, and direct electrical effects. Vasodilator prophylaxis has been utilized for both the prevention and management of coronary spasm [89].

The current limitation of PFA lies in its design, which is optimized for pulmonary vein isolation using basket or lasso catheters. This makes linear ablation or precise targeting of VT substrates challenging. Safety remains a central concern with PFA, particularly the risk of coronary spasm. While prophylactic nitroglycerin (200 micrograms with systolic blood pressure above 80 mmHg) has been shown to mitigate acute vasospasm, evidence of chronic coronary remodeling has emerged: patients undergoing cavotricuspid isthmus ablation with PFA demonstrated ~10% luminal reduction at three months on optical coherence tomography, findings that parallel animal data [90]. Whether preexisting coronary disease amplifies this risk is uncertain, underscoring the need for further evidence before widespread adoption in ischemic VT. At the same time, PFA near the great cardiac vein has shown promise for left ventricular summit arrhythmias. When delivered ≥5 mm from coronary vessels with prophylactic intracoronary nitroglycerin, it can abolish arrhythmias refractory to radiofrequency ablation without inducing spasm, and animal studies confirm the creation of intramural lesions 6–8 mm in depth [91]. Taken together, these observations suggest that routine coronary angiography at the intended ablation site, coupled with follow-up imaging, is prudent, and that longer-term data beyond three months are essential. Ultimately, the safety profile of PFA appears to vary by anatomical location, with surrounding tissue characteristics playing a critical role. Emerging alternative PFA platforms may influence the safety profile of this technology, as differences in energy delivery, catheter design, and waveform characteristics could translate into variable effects on surrounding tissues and coronary vasculature. The lattice-tip catheter, which integrates RF and PFA energy, has shown promising registry outcomes in ventricular arrhythmia ablation, with acute success rates of 81–100% and major complications around 6% [92].

From an efficacy standpoint, several principles familiar from RF ablation remain relevant. In swine ventricular models, lesion depth increased with greater contact force and higher pulse counts, rather than temperature or impedance changes, highlighting the importance of electrode-tissue contact [82, 93]. Transient conduction zones—akin to reversible RF lesions—were also noted, necessitating careful catheter orientation and repeated applications to ensure durable tissue death. This has direct implications for creating continuous linear lesions and avoiding gaps that could sustain arrhythmias. Research into real-time ablation indices may soon enable clinicians to titrate force more precisely with PFA. Ultimately, the goal is irreversible electroporation, yet current mapping and electrogram tools may not reliably distinguish reversible stunning from permanent injury. Animal studies suggest acute electrogram changes within 30 minutes could help predict lesion durability [94]. Developing robust mapping strategies to avoid overestimating electrically silent but viable tissue will be critical for advancing PFA in VT ablation [95].

Given these uncertainties, PFA remains investigational for VT ablation and is not yet widely adopted in routine clinical practice [87]. Notably, Field Medical’s FieldForce™ system is the first contact force-enabled PFA platform specifically designed for VT, demonstrating full-thickness lesions and a reported 78% freedom from VT. While isolated case reports have documented acute success using other PFA systems for VT [96], long-term efficacy and safety data are still lacking.

### 5.3 Stereotactic Body Radiation Therapy for VT Ablation 

Stereotactic body radiation therapy (SBRT), also known as stereotactic arrhythmia radioablation (STAR), represents a novel non-invasive therapeutic approach for patients with refractory VT who are deemed unsuitable for conventional catheter ablation due to hemodynamic instability or intolerance to prolonged procedures under general anesthesia. This cohort, often excluded from standard interventions, underscores a critical unmet need for rapid, non-invasive ablation strategies tailored to the management of life-threatening VAs in severely ill or high-risk populations.

SBRT delivers high-dose radiation to arrhythmogenic myocardial substrates using advanced non-invasive mapping techniques such as multielectrode body surface electrocardiography or electrocardiographic imaging (ECGI), integrated with conventional imaging modalities including cardiac CT, MRI, or fluorodeoxyglucose positron emission tomography (PET). This approach incorporates respiratory and cardiac motion compensation to ensure precise target delineation [97]. The therapeutic effect is partially mediated through late-onset myocardial fibrosis induced by single-fraction doses of 25–35 Gy, aimed at disrupting the VT circuit. Cellular injury mechanisms include DNA damage-induced apoptosis, reactive oxygen species generation, and microvascular disruption [97, 98]. Given its delayed onset of action, SBRT is unsuitable for acute interventions such as VT storm and should be evaluated within an appropriate temporal framework distinct from conventional ablation modalities.

An inaugural case series [99] reported the use of STAR in five high-risk patients with refractory VT, treated with a single 25 Gy fraction while awake. The outcomes were striking, demonstrating a 99.9% reduction in VT episodes—from 6577 to just 4 within six weeks post-ablation—with minimal adverse effects, including mild inflammatory changes in adjacent pulmonary tissue and an average treatment duration of under 15 minutes per patient. Subsequently, the ENCORE-VT trial [100], a single-arm prospective study from a single center, provided pivotal data on the safety and efficacy of STAR. VT burden was significantly reduced (from 119 to 3 episodes at six months after treatment), though notable complications emerged: asymptomatic pericarditis or pericardial effusion in 5 of 19 patients, grade 2 radiation pneumonitis akin to that observed in SBRT-treated lung cancer, and three deaths attributed to recurrent VT. These findings underscore persistent limitations that currently confine STAR to a bail-out therapeutic role. First, the use of ECGI for VT mapping remains investigational, with limited validation and restricted availability across institutions. Second, STAR predominantly targets anatomical substrates, with limited capacity to address dynamic, functional components of VT circuits. This may result in suboptimal treatment, risking both undertreatment and proarrhythmic outcomes due to the emergence of new VT pathways [101]. A recent meta-analysis and systematic review [102] encompassing registered prospective clinical studies from 2016 to 2022 demonstrated that STAR significantly reduces VT burden. However, a high recurrence rate of 79% at one year was noted. Whether this reflects the severity of underlying cardiomyopathy, the extent of arrhythmogenic substrate, or limitations in therapeutic efficacy remains uncertain, particularly given the significant risk of bias detected in the included studies.

Despite its conceptual appeal, STAR presents several limitations and unresolved challenges. First, while ECGI can localize epicardial exit sites during VT, it may inadequately capture endocardial or intramural substrates, potentially missing critical isthmuses within the VT circuit. Second, the precision of focused radiation may be insufficient for patients with diffuse or extensive arrhythmogenic substrates. Third, consensus regarding optimal dosing and treatment frequency is lacking. Fourth, the long-term cardiotoxic effects of radiotherapy—impacting conduction tissue, valvular structures, coronary vasculature, and the pericardium—remain poorly characterized and warrant further longitudinal investigation. Lastly, the technical complexity and reliance on specialized radio-oncology expertise restrict STAR’s implementation to a limited number of highly experienced centers.

At present, STAR should be considered a salvage therapy for patients who are ineligible for conventional catheter ablation due to clinical instability or contraindications. Advancing this modality will require robust interdisciplinary collaboration between cardiac electrophysiology and radiation oncology to refine targeting strategies, optimize safety, and improve therapeutic outcomes [101].

### 5.4 Radiofrequency Ablation With Retractable Needle-Tipped Catheter

This is an advanced technique employing a specialized catheter equipped with an electrically active, extendable and retractable needle (27-gauge, 11 mm in length). This catheter features central lumen that facilitates fluid irrigation, intramural electrogram recordings, and pacing capabilities [103, 104], while also enabling simultaneous ablation [105]. Electrogram recordings and pacing can be performed in both unipolar and bipolar configurations. Unipolar signals are recorded directly from the needle tip, whereas bipolar signals are captured between the needle and a ring electrode located 4.5 mm proximally. When the catheter is oriented perpendicularly within the cardiac chamber, this setup yields a semibipolar configuration, which theoretically reduces susceptibility to wavefront direction and minimizes far-field signal interference [103]. Radiofrequency energy can be delivered through the needle, and the use of ionic media such as saline enhances tissue conductivity (Fig. 5) [67]. This augmentation improves electrogram visualization, particularly in scarred myocardium, and contributes to thermal regulation, thereby reducing the risk of steam pops during ablation [106]. The catheter’s architecture enables integrated functionality—including mapping, pacing, and ablation—comparable to that of conventional ablation catheters.

Initial clinical experience with this technique was reported in a cohort of eight patients with intramural VT substrates [104]. All patients achieved termination of at least one VT episode, with post-procedural non-inducibility. The procedure was well tolerated, with no significant increase in life-threatening complications such as pericardial bleeding or intramural hematoma. Further evaluation of lesion characteristics produced by needle ablation remains ongoing. Schaeffer *et al*. [107] described outcomes in 25 patients undergoing intramural VT ablation using this approach. Their study highlighted the variable and often unpredictable distribution of contrast within myocardial tissue, which correlated with lesion formation. Notably, catheter instability and needle dislodgement were shown to influence lesion formation, as evidenced by variability in contrast stain size, shape, and distribution patterns observed in the study. The presence of contrast staining may serve as a surrogate marker for effective ablation, although this requires further validation.

To enhance lesion depth and uniformity, saline-enhanced radiofrequency can also be integrated with the needle-tipped catheter [108]. This involves the infusion of heated saline directly into the myocardium, promoting convective heat transfer and expanding the ablation zone. While promising, additional research is warranted to optimize this technique for intramural VA ablation and to mitigate the risk of collateral tissue injury.

### 5.5 Miscellaneous Techniques 

In addition to conventional approaches, several novel ablation modalities employing specialized tools have been described in the literature. These include the use of a high-intensity ultrasound ablation catheter [109], RF energy delivered via a 0.014-inch Vision guidewire (Biotronik SE & Co. KG, Berlin, Germany) [110], and the Stingray LP Coronary CTO Re-Entry System (Boston Scientific, Marlborough, MA, USA) [111]. These emerging techniques demonstrate promising efficacy in the ablation of intramural VAs, particularly by enabling innovative strategies for delivering RF energy to deep myocardial substrates. However, their implementation necessitates specialized equipment and procedural expertise that may not be routinely available or widely adopted among cardiac electrophysiologists. Nonetheless, these methods merit consideration as alternative therapeutic options, especially in collaboration with interventional cardiology teams.

## 6. Integration of Cardiac Imaging in VT Ablation Strategy

Pre-procedural cardiac imaging—particularly cardiac MRI and CT—allows operators to define the VT substrate before entering the lab, enabling more efficient procedural planning, especially in non-ischemic cardiomyopathy [112]. These patients often have heterogeneous substrates or mixed ischemic components, making ablation more challenging and more dependent on imaging guidance. Substrate patterns vary by etiology, and the presence of mid-myocardial or epicardial involvement often necessitates additional tools or advanced lesion-creation techniques [113]. During the procedure, electrogram characteristics and entrainment maneuvers further clarify how these substrates participate in the VT circuit. Scars exceeding 25% of myocardial thickness frequently harbor the circuit, and their distribution influences both ablation strategy and prognosis [114]. For example, anteroseptal scars are associated with higher recurrence and greater procedural risk due to the difficulty of achieving adequate lesion depth near the conduction system and vascular structures [114, 115], often requiring techniques such as ethanol infusion or bipolar ablation. Epicardial access may also be needed, particularly for inferolateral scar–related VT [114]. Overall, pre- and periprocedural imaging provides essential visualization of arrhythmogenic substrates, guiding the selection of optimal lesion-delivery strategies and contributing to shorter procedures and lower recurrence rates.

## 7. Conclusions & Future Directions

**Fig. 6.**
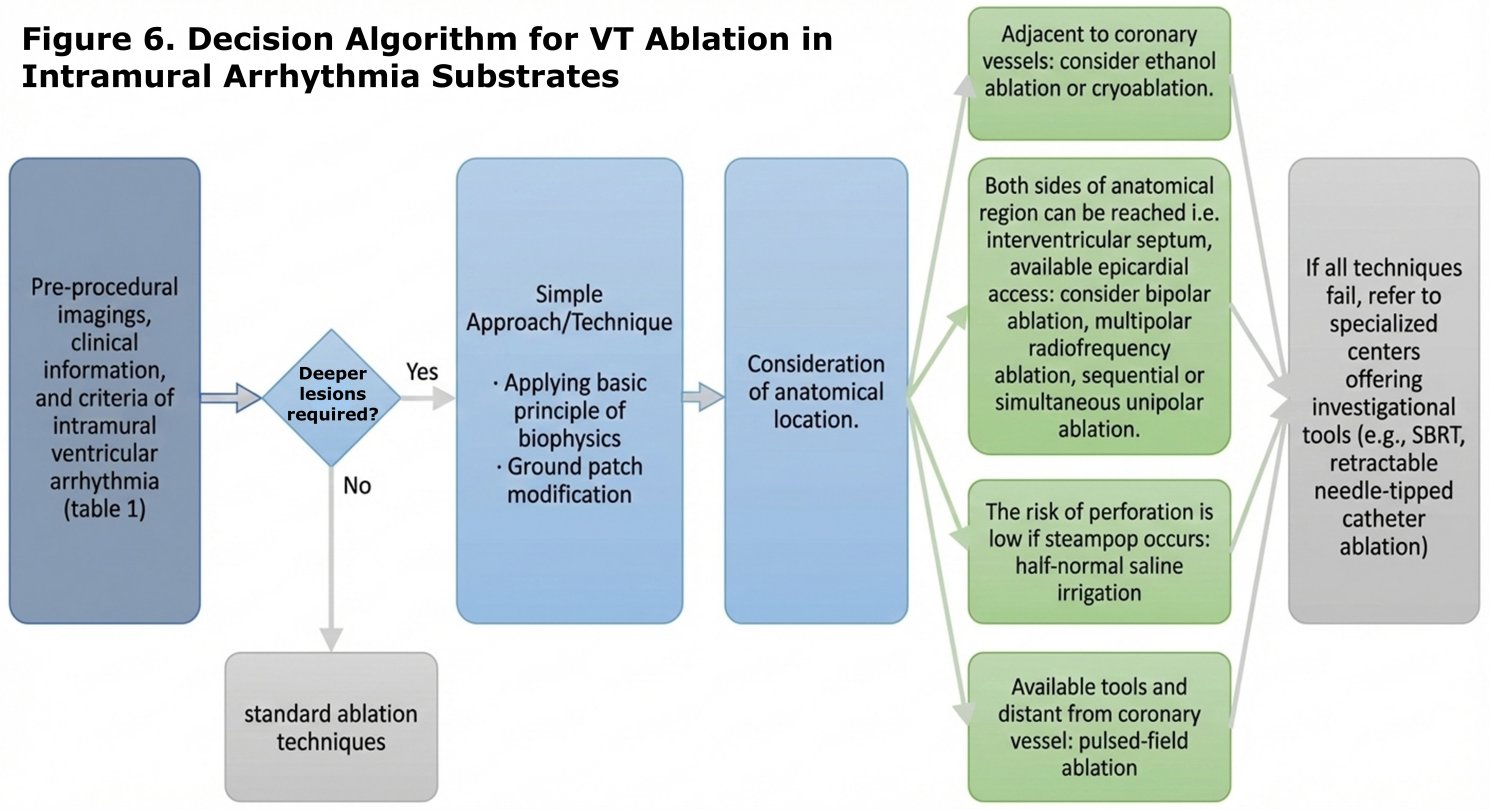
**Decision making algorithm for VT ablation in intramural arrhythmia substrates**.

The management of VAs arising from intramural substrates presents a formidable challenge, particularly in patients with advanced structural heart disease. Conventional catheter ablation techniques often fall short in achieving durable effective lesions, especially when arrhythmogenic substrates reside deep within the myocardial wall. This review has explored a spectrum of emerging strategies aimed at enhancing lesion depth. Each modality offers unique advantages in substrate access and lesion formation, yet none of them singularly addresses the full complexity of intramural VT circuits. We propose a stepwise approach for selecting the appropriate method, prioritizing ease of application in the laboratory based on technical feasibility (Fig. 6). The evolution of these techniques underscores a paradigm shift toward substrate-specific and anatomy-guided ablation strategies. Integration of advanced imaging modalities—such as cardiac MRI, PET, and ECGI—has enabled more precise localization of arrhythmic substrates, while novel energy delivery systems continue to extend the boundaries of lesion depth and transmurality. Looking forward, the development of personalized ablation protocols tailored to individual myocardial architecture and substrate characteristics represents a promising frontier. In conclusion, the field is poised to redefine therapeutic paradigms for intramural VT and improve outcomes in this challenging population.

## Abbreviations

AI, ablation index; CF, contact force; CT, computed tomography; ECGI, electrocardiographic imaging; gs, gram-seconds; HNS, half-normal saline; FTI, force-time integral; MRI, magnetic resonance imaging; PFA, pulsed-field ablation; RF, radiofrequency; SBRT, stereotactic body radiation therapy; STAR, stereotactic arrhythmia radioablation; ULTC, ultralow-temperature cryoablation; VA, ventricular arrhythmia; VT, ventricular tachycardia.
